# Effects of Sludge Retention Time on the Performance of Anaerobic Ceramic Membrane Bioreactor Treating High-Strength Phenol Wastewater

**DOI:** 10.1155/2020/8895321

**Published:** 2020-08-01

**Authors:** Chunhua He, Chuanhe Yang, Shoujun Yuan, Zhenhu Hu, Wei Wang

**Affiliations:** ^1^Department of Municipal Engineering, School of Civil Engineering, Hefei University of Technology, Hefei 230009, China; ^2^Anhui Provincial Engineering Laboratory for Rural Water Environment and Resources, Hefei 230009, China

## Abstract

Anaerobic ceramic membrane bioreactor (AnCMBR) is an attractive alternative for the treatment of high-strength phenol wastewater, but the effects of sludge retention time (SRT) on the performance and membrane fouling are still unclear. The results indicated that the AnCMBR was successfully employed to treat high-strength wastewater containing 5 g phenol L^−1^. The removal efficiencies of phenol and chemical oxygen demand (COD) reached over 99.5% and 99%, respectively, with long SRT and short SRT. SRT had no obvious effect on the performance of the AnCMBR treating high-strength phenol wastewater with long time operation. The strong performance robustness of AnCMBR benefited from the enrichment of hydrogenotrophic methanogens and syntrophic phenol-degrading bacteria. However, the decline of SRT led to a more severe membrane fouling in the AnCMBR, which was caused by the small size of sludge flocs and high concentration of protein in the biopolymers. Therefore, this work presented a comprehensive insight to the feasibility and robustness of the AnCMBR for treating high-strength phenol wastewater.

## 1. Introduction

Many coal industrial liquid effluents, such as coking and coal gasification, contain a very high concentration of phenolic compounds [1, 2]. For example, the concentration of phenolic compounds in coal gasification wastewater varies from 4.5 to 7.5 g L^−1^ [3]. Although both the anaerobic and aerobic processes were used to treat the phenolic wastewater, the anaerobic process was a more attractive alternative because of its advantages of low operation cost and energy resource recovery [4, 5]. It is a challenge for anaerobic process to treat high-strength phenol wastewater, since the anaerobic sludge is difficult to be granulated and easy to escape from the bioreactor under strong toxicity condition [6].

Anaerobic membrane bioreactor (AnMBR) is a promising alternative for the treatment of industrial wastewaters. AnMBR with combination of anaerobic digestion and membrane separation endows some advantages, such as high sludge concentration, low sludge yield, and excellent removal capacity [7, 8]. Therefore, the AnMBR can be used to treat the high-strength phenol wastewater, because sufficient amount of biomass remained in the reactor which could overcome the slow hydrolysis rate of phenol [9]. However, membrane fouling is one of the biggest obstacles which limited the application of AnMBR in wastewater treatment [10, 11]. A feasible alternative to alleviate membrane fouling was to use the ceramic membranes as a replacement of polymeric membranes due to their higher membrane hydrophilicity [12, 13]. Ceramic membrane filtration operated better than polymeric membrane in terms of lower fouling rate, stronger performance robustness against chemical exposure, and higher mechanical strength [14]. It provided a possibility for the treatment of high-strength phenol wastewater using ceramic membranes in the anaerobic reactor. However, the corresponding operational parameter and its effects on the anaerobic ceramic membrane bioreactor (AnCMBR) treating high-strength phenol wastewater are still unclear.

As known, the sludge retention time (SRT) is a feasible operational parameter of bioreactor which directly affects treatment performance and membrane fouling [15]. Nevertheless, the views of previous studies on the SRT effect on the treatment performance and the membrane fouling were contradictory. It was widely considered that better digestion efficiency and effluent quality and higher methane yield could be achieved at the longer SRT [16]. However, the effects of SRT on the membrane fouling of the AnMBR treating different wastewater were different. Han et al. found that the longer SRT resulted in more serious membrane fouling because the sludge particles were more severely deposited on the membrane surface and extracellular polymeric substances (EPS) of sludge would be increased at longer SRT [17]. But, Estrada-Arriaga and Mijaylova found that a lower SRT and hydraulic retention time (HRT) caused more serious membrane fouling when the MBR was operated with treating estrogen-containing wastewater [18]. Therefore, the effect of SRT on the robustness performance of AnCMBR should be explored which was practically significant for the treatment of high-strength phenol wastewater.

The objectives of this study were to examine the influence of SRTs on the performance robustness of AnCMBR treating high-strength phenol wastewater. The effect of SRTs on the transmembrane pressure (TMP) of AnCMBR was presented. Moreover, the soluble microbial products (SMP), EPS, the particle size distribution (PSD), and microbial community structure of sludge at different SRTs were investigated.

## 2. Materials and Methods

### 2.1. Experimental Setup and Synthetic Wastewater

**Figure 1:**
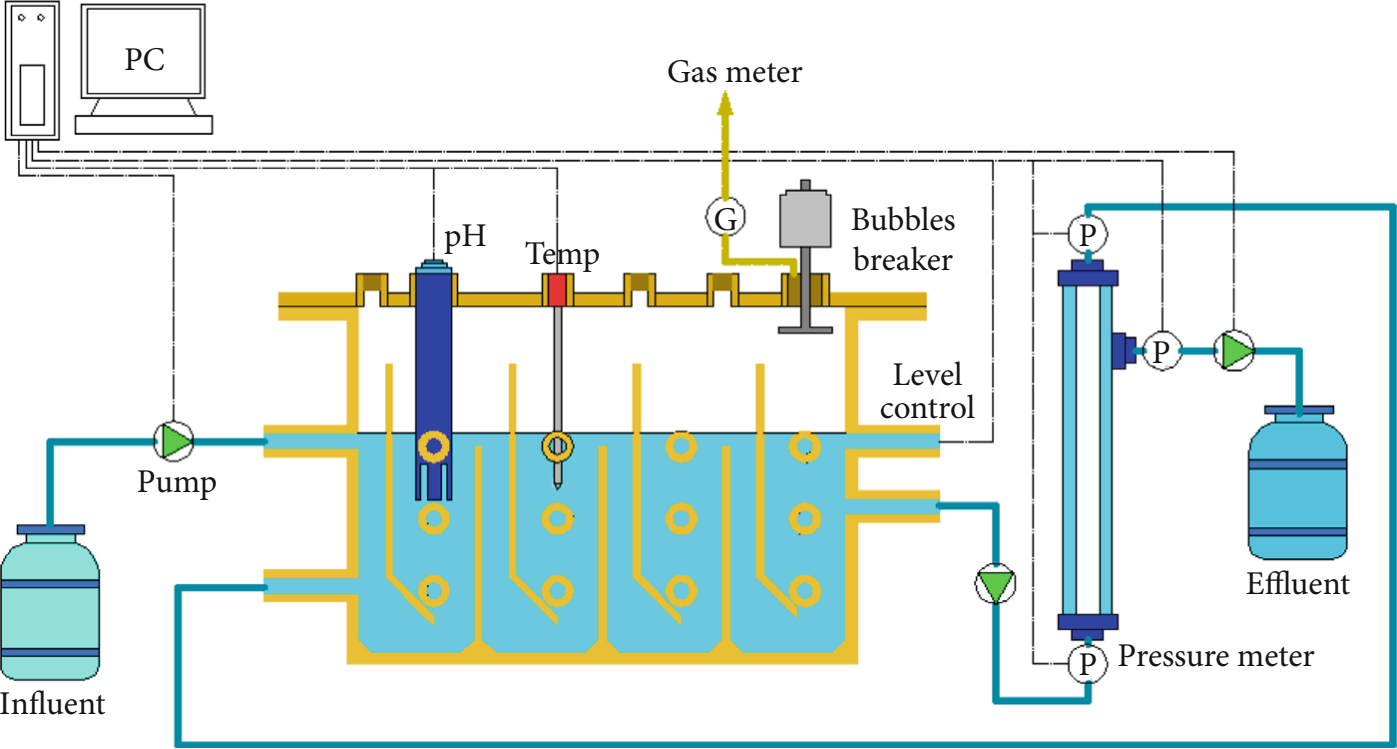
The schematic diagram of the AnCMBR.

The schematic diagram of the AnCMBR is shown in Figure 1 and the details were described in a previous study [19]. The volume of the reactor was 6.2 L with a hydraulic retention time of 2 days. The whole experiment could be divided into two phases (phase I and II), and the longer SRT was controlled at 233 days at phase I and the shorter SRT was controlled at 61 days at phase II. Before this study, the inoculation sludge was acclimated to phenolic wastewater (5000 mg phenol L^−1^) for about one month. The mixed liquor suspended solids (MLSS) and mixed liquor volatile suspended solids (MLVSS) concentrations of the inoculum was 18.53 g L^−1^ and 12.90 g L^−1^, respectively. The wastewater consisted of phenol (5000 mg L^−1^) and sodium acetate (2770 mg L^−1^), which contributed to the total COD concentration of around 14000 mg L^−1^. Additionally, the macronutrients, micronutrients, yeast extract, and a phosphate buffer solution were also added, and their concentration and composition were referred to a previous study [19].

### 2.2. Effects of SRTs on the Transmembrane Pressure (TMP) of AnCMBR

The transmembrane pressure (TMP) was detected in real time by the pressure sensors, and the LabVIEW software was used for recording the data. The fouled membrane in the AnCMBR was physically and chemically cleaned at the end of phases I and II. The physical cleaning was applied by scrubbing cake layer from the membrane surface using tap water. After that, the chemical cleaning was conducted to remove irreversible fouling by soaking the membrane in NaClO solution (0.5%) for 4 hours and followed by flushing with tap water [20].

### 2.3. Effects of SRTs on SMP and EPS of Sludge

At the end of every phase, the sludge samples were collected for the analyses of SMP and EPS. The sample supernatant was used to analyze the concentration of SMP, which was obtained after centrifugation with 9000 r min^−1^for 15 min and filtration with 0.45 *μ*m filter. The cation exchange resin (CER) technique was adopted to extract EPS [21]. The concentrations of carbohydrates and protein were measured by phenol-sulphuric acid method [22] and a modified version of the Lowry method [23], respectively. The concentrations of carbohydrates and protein were detected by a spectrophotometer at absorbance of OD_490_ nm and OD_750_ nm.

### 2.4. Effects of SRTs on Microbial Community Structure

At the end of every phase, the sludge samples were collected for the analysis of microbial community structure. A bacterial genomic extraction kit (E.Z.N.A. Mag-Bind Soil DNA Kit, OMEGA) was employed to extract DNA from the sludge samples, and the agarose gel electrophoresis was used to check the integrity of DNA. The primers of 341F (5′-CCTACGGGNGGCWGCAG-3′)/805R (5′-GACTACHVGGGTATCTAATCC-3′) were selected for amplifying of bacterial populations, and the primers of 340F (5′-CCCTAYGGGGYGCASCAG-3′)/1000R (5′-GGCCATGCACYWCYTCTC-3′) were employed for amplifying of *archaea* populations. After PCR reaction, DNA purification, and quantification, the sludge samples were sequenced by Illumina MiSeq platform (Illumina, Inc., San Diego, CA, USA). The above analytical procedures were conducted by Shanghai Sangon Biological Engineering Technology and Services Co., Ltd. The analysis of sequence data was operated as the previous literature [24].

### 2.5. Other Analytical Methods

The concentration of phenol was determined by high-performance liquid chromatography (1260 Infinity, Agilent, USA) with a mobile phase of 50% acetonitrile. A laser granularity distribution analyzer (Malvern Instruments, MS-2000) was used to analyze the particle size distribution (PSD) of sludge samples. The concentrations of COD, MLSS, and MLVSS were measured according to the standard methods [25].

## 3. Results and Discussion

### 3.1. Effects of SRTs on the Treatment Performance of AnCMBR Treating High-Strength Phenol Wastewater

**Figure 2:**
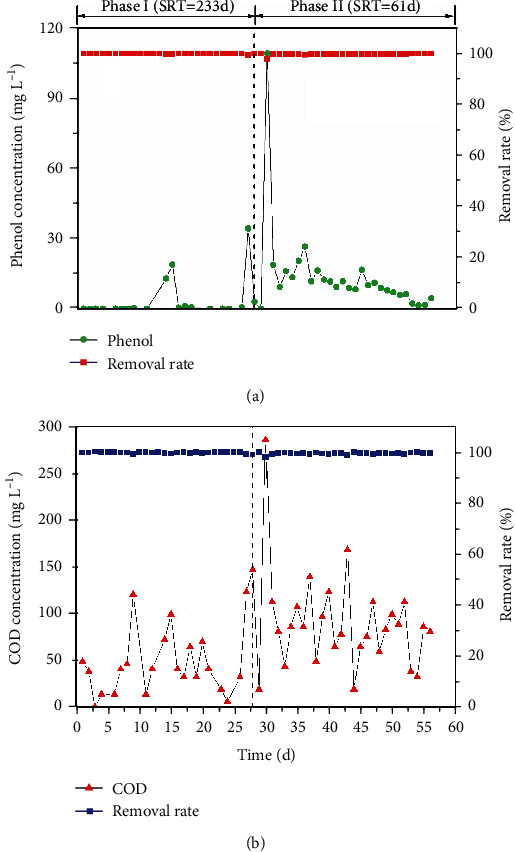
Treatment performance of AnCMBR treating high-strength phenol-containing wastewater with different SRTs (phase I: SRT, 233 days; phase II: SRT, 61 days).

Figure 2 shows the treatment performance of AnCMBR treating high-strength phenol wastewater at two different SRTs. According to Figure 2(a), when the SRT was 233 days in phase I, the phenol concentration was not detected in the effluent. Subsequently, the SRT was decreased to 61 days in phase II, and the effluent became worse in the first five days in which the phenol concentration was about 85.3-138.7 mg L^−1^. The treatment performance gradually normalized, and the removal efficiency of phenol was about 99.8%. As shown in Figure 2(b), the removal efficiency of COD remained around 99.8% and 99.4% with longer SRT and shorter SRT, respectively. An obvious fluctuation of effluent was also observed when SRT was decreased at the early stage of phase II (shorter SRT) in which the COD concentration in the effluent was higher to 285.33 mg L^−1^. Accompanied with the running, the effluent COD concentration declined to lower than 100 mg L^−1^. The results suggested that the AnCMBR had strong performance robustness for treating high-strength phenol wastewater. Due to the shock loading of high-strength phenol, the UASB reactor was very difficult to acquire a strong performance robustness [26]. Previous literature demonstrated that 2000 mg L^−1^ of phenol caused a remarkable inhibitory effect on the phenol degraders and methanogens in the saline UASB reactor [24]. When anaerobic bacteria were exposed to high concentration of phenol precipitately, the conversion of phenol to methane was blocked, resulting in the accumulation of phenol in the effluent [24]. The AnMBR was particularly suitable for high-strength wastewater due to its long SRT and sufficient amount of biomass, thus facilitating higher organic loading and performance stability [27, 28]. For the high-strength phenol wastewater, the slow-growing phenol degraders and methanogens could be enriched in the AnCMBR. The well-cultivated phenol degraders and methanogens in the AnCMBR should play a key role to remain high metabolic activity under extremely high concentration of phenol. Therefore, the specialized anaerobic microorganisms might be strengthened in the AnCMBR under the extremely high concentration of phenol. As calculated, phenol loading rate of sludge was correspondingly enhanced from 0.2 (longer SRT) to 0.275 (shorter SRT) g phenol g^−1^ MLVSS d^−1^, and it was much higher than the reported values [24, 29]. The strong performance robustness of AnCMBR treating high-strength phenol wastewater was mainly attributed to the efficient microbial community [30, 31]. As shown in Figure 2, it was also presented that the worse treatment performance and the obvious fluctuation of effluent were observed at the early stage of SRT decreasing. The worse performance with shorter SRT might contribute to the decrease of sludge concentration and the shift of microbial community structure in the AnMBR [30]. The bacteria and archaea communities gradually adapted the high-strength phenol condition and then the treatment performance was recovered [31]. Therefore, the AnCMBR was a promising alternative to treat high-strength phenol wastewater.

### 3.2. Effects of SRTs on the Transmembrane Pressure (TMP) of AnCMBR

**Figure 3:**
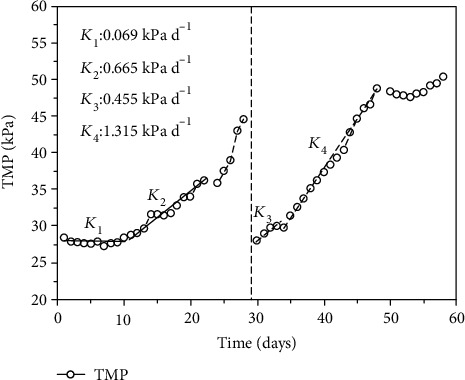
The effect of SRTs on the TMP variations of AnCMBR.

Figure 3 shows the TMP variation of AnCMBR at the two different SRTs. It could be found that the TMP values of shorter SRT increased faster than that of longer SRT. Despite the initial TMPs were 28.40 and 28.05 kPa with two different SRTs, but it took 28 and 16 days to reach a TMP of 45 kPa with longer SRT (phase I) and shorter SRT (phase II), respectively. The TMP profiles were composed of two phases: horizontal and exponential increase [12, 32]. At the horizontal increase phase, the ascending rate was 0.069 kPa d^−1^ with the duration of 12 days at phase I (longer SRT), while the increasing rate was up to 0.455 kPa d^−1^ with the duration of only 4 days at phase II (shorter SRT). The horizontal increase of TMP might result from the gradually accumulated organic macromolecules, microorganisms, and soluble compounds on the membrane surface, which did not significantly reduce the membrane flux [33]. At the exponential increase phase, the slopes of TMP profiles were 0.665 and 1.315 kPa d^−1^ at phases I and II, respectively. The exponential increase of TMP chiefly ascribed to the plugged membrane pores by the microbial products and thick cake layer on the membrane surface, which aggrandized the fouled membrane areas so that the membrane flux rose sharply [34]. It indicated that both the horizontal and exponential increase rates of TMP with shorter SRT were higher than that of longer SRT and the results were different from the previous study. Jeison and van Lier found that membrane fouling was accelerated by the high sludge concentration in the AnMBR with long SRT [35]. Huang et al. explored the role of SRTs on the performance of submerged AnMBR for domestic wastewater treatment and observed that smaller particle size and less particle flocculation which resulted from lower concentrations of protein and carbohydrate in EPS accelerated the fouling development at longer SRT [33]. However, Huang et al. reported that the higher concentration of SMP at a shorter SRT also significantly affected membrane fouling [36]. In this study, the shorter SRT caused the more severe membrane fouling in AnCMBR with treating high-strength phenol wastewater. The reason might be related to the change of microbial products (e.g., EPS and SMP) and the particle size of sludge under the extreme high-strength phenol condition.

### 3.3. Effects of SRTs on the Soluble Microbial Products (SMP) and the Extracellular Polymeric Substances (EPS) of Sludge

**Figure 4:**
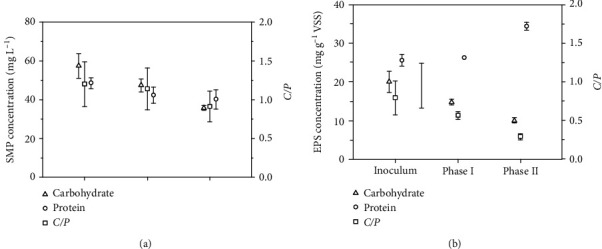
The effects of SRT on the SMP and EPS composition of sludge. (a) SMP compositions with two different SRTs. (b) EPS compositions with two different SRTs (phase I: SRT, 233 days; phase II: SRT, 61 days; *C*/*P*: mass concentration ratio of carbohydrate to protein).

Figure 4 shows the SMP and EPS composition at the two different SRTs. SMP and EPS which mainly contain proteins and carbohydrates were reported to play important roles in the membrane fouling [33]. As shown in Figure 4(a), the proteins of SMP declined from 42.3 ± 4.1 to 40.0 ± 4.9 mg L^−1^ with SRT shortening, and the carbohydrates of SMP also declined from 47.3 ± 3.4 to 35.7 ± 1.2 mg L^−1^. The result indicated that SMP which included proteins and carbohydrates decreased with shorter SRT. Furthermore, the ratio of carbohydrates to protein (*C*/*P*) of SMP significantly decreased with a shorter SRT, which attributed to the remarkable decline of carbohydrate concentration and the little deccrease of protein concentration in the SMP. This result was consistent with the previous study which reported that the microorganisms metabolized more actively and resulted in more organic compounds degraded and less SMP remained with a shorter SRT [33]. Our findings indicated that the severe membrane fouling with the shorter SRT was not caused by the SMP variation.

Figure 4(b) shows the variation of EPS concentration at the two different SRTs. Similar to the SMP, a significant decline of carbohydrates and *C*/*P* ratios were found in EPS with the decrease of SRT. However, the concentration of protein in EPS increased from 26.3 ± 0.4 to 34.4 ± 1.0 mg g^−1^ VSS^−1^ with the enhancement of phenol loading. The results indicated that the increase of phenol loading facilitated the production of protein in EPS. It was known that EPS which was generated by bacterium and enveloped the cells against the stress conditions [37]. As the phenol loading increased with shorter SRT, the microbial communities faced more toxic condition; hence, the protein of EPS increased greatly. The result was consistent with the previous study in which the protein concentration of EPS raised with the increase of phenol concentration in the anaerobic reactor [38]. EPS played an important role in the degradation of phenol, which was absorbed firstly and further be degraded by the relevant enzymes in EPS [39]. Therefore, the increase of protein concentration in EPS was closely related to the treatment performance of the AnCMBR.

In addition, the protein and carbohydrates were the main components of SMP and EPS, but the carbohydrates could be considered hydrophilic, while many proteins had hydrophobic properties [40]. The decrease of *C*/*P* ratio resulted in a decrease in the negative charge of the sludge surface, thereby increasing the surface hydrophobicity. Lee et al. reported that hydrophobicity of sludge was connected with adhesion forces [41]. Furthermore, the hydrophobic foulants could cause greater adhesion to hydrophobic membranes [42]. Hence, the increase of protein concentration in EPS might be responsible for the increasing adhesion forces and exponential increase in membrane fouling at the shorter SRT.

### 3.4. Effects of SRTs on the Particle Size Distribution (PSD) of Sludge

**Figure 5:**
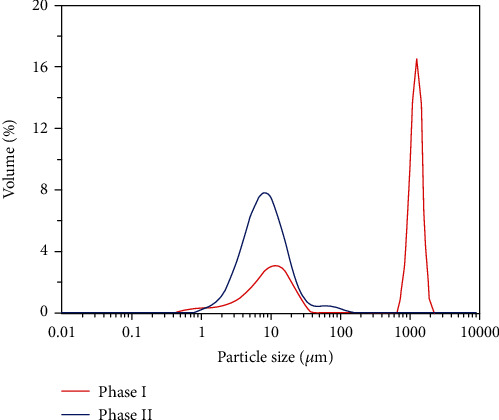
The effect of SRT on the particle size distribution of sludge (phase I: SRT, 233 days; phase II: SRT, 61 days).

The PSD of sludge in the AnCMBR at the two different SRTs is shown in Figure 5. The results suggested that the shorter SRT caused a descending trend of floc size. The curve of PSD exhibited two independent peaks with longer SRTs. The average particle size of larger sludge flocs was 1300 *μ*m with longer SRT, but it was almost not observed with shorter SRT. Furthermore, the particle size of small sludge flocs decreased from 12.0 to 8.6 *μ*m with SRT of 61 days. The large size sludge flocs were disintegrated, which was caused by the increase of phenol loading with shorter SRT. Han et al. reported that the microenvironmental characteristics in different sizes of flocs influenced the bacterial diversity and distribution of functional microbes, thereby the size distribution of sludge flocs played an important role in the removal of pollutants [43]. The decrease of floc size suggested that the microbial community structure might shift to the one with higher mass transfer efficiency and stronger tolerance to the environment. Similarly, the proportion of small size anaerobic granular sludge increased by the increase of phenol loading, corresponding with the shifts in the dominant microbial community [38]. Therefore, the decrease of floc size might be a favorable feedback responding to the shift of community structure and the enrichment of phenol degraders under extremely high-strength phenol wastewater.

As discussed in the previous section, the increase of phenol loading would lead to the decrease of carbohydrate concentration in EPS which was responsible for both adhesion and cohesion interactions between sludge cells [44]. In order to adapt to the change of environment, the sludge flocs trended to be smaller to enhancing mass transfer, enabling the system to adapt the change of OLR and achieving a higher organic removal rate [45]. In addition, the shear stress induced by biogas sparging in the reactor also brought about the decrease of particle size [19]. However, the small sludge flocs would induce denser biocake in the membrane surface. Although ceramic membranes had lower fouling propensity than polymeric membranes due to the weaker bonding between the foulants and membranes [46, 47], the small sludge flocs could plug the membrane pores easily, thereby sharply reducing the duration of horizontal increase of TMP with a shorter SRT. Therefore, higher concentration of protein in EPS and smaller particle size which were caused by a shorter SRT were the two main factors for more severe membrane fouling.

### 3.5. Effects of SRTs on the Change of Microbial Community Structure

**Figure 6:**
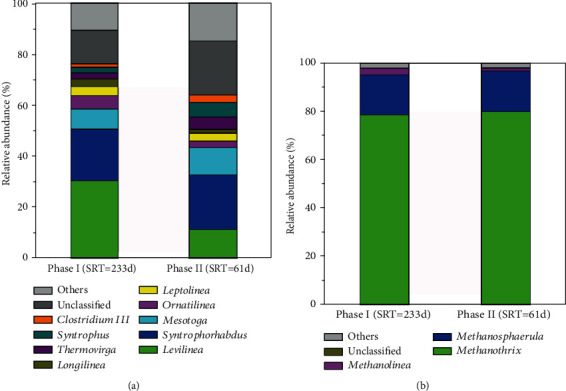
The relative abundance of bacteria and archaea at genus level with different SRTs (a) bacteria, (b) archaea. “Other” represents all classified taxa that were <1% in all samples.

Figure 6(a) shows the relative abundances of the major bacteria at genus level with the two different SRTs. With a longer SRT, the dominant bacterial genera were *Levilinea* (30.257%), *Syntrophorhabdus* (20.23%), *Mesotoga* (7.95%), *Ornatilinea* (5.22%), *Leptolinea* (3.59%), *Longilinea* (3.05%), *Thermovirga* (2.34%), *Syntrophus* (2.21%), and *Clostridium III* (1.30%), adding up to approximately 76.15% of relative abundances of all classified sequences. After switching to a shorter SRT, the dominant bacterial genera were *Levilinea* (11.13%), *Syntrophorhabdus* (21.53%), *Mesotoga* (10.56%), *Ornatilinea* (2.56%), *Leptolinea* (3.10%), *Longilinea* (1.49%), *Thermovirga* (4.90%), *Syntrophus* (5.64%), and *Clostridium III* (3.01%). The highest abundance of *Levilinea* significantly declined as the SRT decreased, which might resulted from the increase of phenol loading with a shorter SRT. In line with the previous study, the relative abundance of *Levilinea* witnessed a drop with the increase of phenol loading in the UASB reactor. *Levilinea* was anaerobic bacteria, which could convert amino acids and sugars into hydrogen and acetic and lactic acids [48]. Meanwhile, the relative abundances of other genera such as *Ornatilinea*, *Leptolinea*, and *Longilinea* decreased with shorter SRT. Contrary, the increase of *Thermovirga* and *Mesotoga* presented stronger tolerance to the increase of phenol loading. The former was able to ferment proteinous substrates, some single amino acids and organic acids and could be enriched with the enhancement of phenol loading [49]. The latter, which belonged to phylum *Thermotogae* and preferred to dwelling in the high salt and mesophilic conditions, could degrade fatty acids and play an essential ecological role in the ecosystems contaminated by aromatic compounds [50, 51]. Genus *Syntrophorhabdus*, which belonged to the class *Deltaproteobacteria*, was the subdominant group of phase I [52]. The role of *Syntrophorhabdus* was identified to convert phenol to benzoate and further to H_2_ and acetate in the syntrophic consortium with a hydrogenotrophic methanogen [53]. It indicated that *Syntrophorhabdus* had a stronger endurance toward the increase of phenol loading than genus *Levilinea* at an extremely high concentration of phenol. *Syntrophus* was one of the faster growing strain, which was reported to have a relatively high abundance in anaerobic treatment of phenolic wastewater [54] and could transform benzoate into acetate and H_2_/CO_2_ [55]. Previous study reported that syntrophic bacteria such as *Syntrophus* and *Syntrophorhabdus* could work solely to convert phenol to acetate [56]. Hence, the two syntrophic bacteria were the dominant phenol degraders for successfully treating extremely high-strength phenol wastewater. Although the removal of phenol was not affected by the decreased SRT, the community structure of phenol degraders was changed, corresponding with *Syntrophorhabdus* and *Syntrophus* exhibiting a strong robustness. However, these slow-growing syntrophic bacteria, which played an important role in degrading phenol under the extremely high concentration of phenol, were easily washed out in the conventional anaerobic reactors. But the increase of *Syntrophorhabdus* and *Syntrophus* with shorter SRT presented an advantage of AnCMBR holding a sufficient amount of biomass in the reactor [39]. Therefore, it indicated that the enrichment of syntrophic phenol-degrading bacteria in the AnCMBR ensured efficient phenol removal and strong performance robustness at an extremely high concentration of phenol.

Figure 6(b) shows the effect of SRT on the relative abundances of *archaea* at genus level. The results indicated that the change of SRT had no obvious effect on *archaea*. The dominant populations were *Methanothrix*, *Methanosphaerula*, and *Methanolinea*, and the relative abundances of *archaea* with two different SRTs were similar. As known, *Methanothrix* was affiliated to acetoclastic methanogens [57] and its relative abundances slightly increased from 78.55% to 79.81% when SRT declined from 233 days to 61 days. A previous study presented that *Methanothrix* was the dominant *archaea* on the surface of granules for treating complex phenolic wastewater in UASB reactors [58]. The findings indicated that acetoclastic methanogenesis was the main methane production way. Despite the increase of phenol loading rate, the relative abundances of *Methanosphaerula* and *Methanolinea* which all belonged to hydrogenotrophic methanogen were not dramatically changed with shorter SRTs [59]. The result indicated that the hydrogenotrophic methanogens were stable in AnCMBR, in spite of high-strength phenol condition. The hydrogenotrophic methanogens played an important role in the conversion of phenol to methane, which could make the benzoate degradation reaction thermodynamically favorable [54]. Due to the enhancement of phenol loading with shorter SRT, the phenol degraders would shift to syntrophic phenol-degrading bacteria with stronger endurance to high-strength phenol condition, but this shift required the assistance from hydrogenotrophic methanogens. Poirier et al. found that the ratio of methane production via the hydrogenotrophic pathway was gradually increased along with the increase of phenol stress, which indicated that hydrogenotrophic methanogen also held strong endurance to extremely high concentration of phenol [31]. Therefore, the hydrogenotrophic methanogens and syntrophic phenol-degrading bacteria should play an important role in the degradation of high-strength of phenol. Although the phenol loading rate increased with a shorter SRT, the syntrophic bacteria such as *Syntrophus* and *Syntrophorhabdus* were enriched and hydrogenotrophic methanogens were kept stable in the AnCMBR. The AnCMBR provided a strong robustness pathway for anaerobic treatment of high-strength phenol wastewater.

## 4. Conclusion

Based on the experimental results, the main conclusions were drawn as follows:

1. The AnCMBR achieved satisfactory treatment performance with phenol concentration of 5 g L^−1^ although phenol loading rate of sludge was increased from 0.2 to 0.275 g phenol g^−1^ MLVSS d^−1^
2. Severe membrane fouling emerged in the AnCMBR at a shorter SRT, resulting from the increase of protein concentration in EPS and the decline of floc size
3. With the increase of phenol loading at a shorter SRT, the enrichment of syntrophic phenol-degrading bacteria and the stability of hydrogenotrophic methanogens might be the main reason of the strong performance robustness of AnCMBR treating high-strength phenol wastewater

## Data Availability

The data used to support the findings of this study are available from the corresponding author upon request.
